# Wrist-Ankle Acupuncture for the Treatment of Pain Symptoms: A Systematic Review and Meta-Analysis

**DOI:** 10.1155/2014/261709

**Published:** 2014-07-15

**Authors:** Li Bing Zhu, Wai Chung Chan, Kwai Ching Lo, Tin Pui Yum, Lei Li

**Affiliations:** ^1^School of Chinese Medicine, The University of Hong Kong, 10 Sassoon Road, Pokfulam, Hong Kong; ^2^Acupuncture and Tui-na Clinical Centre for Teaching and Research, School of Chinese Medicine, The University of Hong Kong, Hong Kong

## Abstract

Routine acupuncture incorporates wrist-ankle acupuncture (WAA) for its analgesic effect, but WAA is not widely used in clinics due to incomplete knowledge of its effectiveness and concerns about less clinical research and because less people know it. This study aimed to assess the efficacy and possible adverse effects of WAA or WAA adjuvants in the treatment of pain symptoms. This study compared WAA or WAA adjuvant with the following therapies: western medication (WM), sham acupuncture (SA), or body acupuncture (BA). Randomized controlled trials (RCTs) were searched systematically in related electronic databases by two independent reviewers. 33 RCTs were finally included, in which 7 RCTs were selected for meta-analysis. It was found that WAA or WAA adjuvant was significantly more effective than WM, SA, or BA in pain relief. There was nothing different between WAA and SA in adverse events, but WAA was marginally significantly safer than WM. Although both WAA and WAA adjuvant appeared to be more effective than WM, SA, or BA in the treatment of pain symptoms with few side effects, further studies with better and more rigorously designed are still necessary to ensure the efficacy and safety issue of WAA due to the poor methodology and small sample size of previous studies.

## 1. Introduction

Today, pain represents an increasingly common public health problem in the world. In 2001, the International Association for the Study of Pain defined pain as tissue damage or potential tissue damage that causes unpleasant sensory and emotional experience [1]. According to a report from the American Medical Research Institute in 2011, there were at least one hundred million people who suffered from acute and chronic pain in the United States. The report also predicted chronic disability caused by back pain will reach 1% of the United States adults. Medical costs and loss of productivity due to pain directly affected the economy, which was estimated to cost 560 to 630 billion dollars. With an aging population, this serious problem of chronic pain in the elderly will continue to increase in US [2]. Not only is pain prevalent in United States, but it is also becoming more common in Europe and Asia [3]. The substantial number of people suffering from pain, the great medical costs, and the negative impact of pain on the quality of life are all reasons that the treatment of pain deserves greater attention. As the international authorities in pain Melzack and Wall said, “Pain is a major problem which has no national boundaries, solving the problem needs the joint efforts of the world” [4].

Nearly forty years ago, acupuncture became a hot topic in western countries and began its international fame ever since the publication of the article* “Now Let Me Tell You about My Appendectomy in Peking"* by James Reston, whose serve abdominal pain was relieved by acupuncture in one hour [5]. An increasing number of western scholars began to pay attention to the relationship between pain and acupuncture, and patients suffering from pain in western countries also began to choose acupuncture as a therapeutic method [6]. As scholars in China and abroad dedicated themselves to discover whether acupuncture is effective for the treatment of pain, innovations in routine acupuncture surfaced through the decades. One such innovation was WAA, a new subcutaneous acupuncture developed by Professor Zhang Xinshu in the 1970s. Although the needle positions are only located in the wrist and ankle, WAA treats a range of problems throughout the body, especially pain symptoms. Compared with Routine Acupuncture, WAA does not need to appear “Deqi” and its needle positions are located only in the wrists and ankles where no important organs and vessels are located. Moreover, WAA does not need to follow the traditional Chinese theory “therapy with syndrome differentiation” and only requires precise needle locations appropriate to the patients' signs and symptoms. Hence in comparison with Routine Acupuncture, WAA is a relatively safe, convenient, and quick procedure [7].

From the perspective of Traditional Chinese Medicine theory, some scholars hold that the analgesic mechanism of WAA is derived from the cutaneous regions theory of Huangdi's Inner Classic which means the distribution areas of twelve cutaneous regions are similar to the body surface distribution area of the twelve meridians. WAA adjusts the function of corresponding meridians and viscera through stimulating cutaneous regions, making blood running unobstructed which is so called “There is not pain when the circulation of Qi and blood is unobstructed”. Much of the Traditional Chinese Medicine theory is abstract just like arts and it seems to be difficult to understand if you do not have an educational background of traditional Chinese medicine. Just as Liu Liang said, western medicine depends on science to create and assess drugs at the molecular level. In Asia, there is a commonly held belief that there is an art to healing too, and that both art and science should cooperate to help eradicate illness and relieve suffering [8]. Now there are many scholars dedicated to study the analgesic mechanism of WAA from the perspective of modern science. And someone found that skin does exist the distribution of meridians. In that study, radiation nuclide high acid sodium was injected into participants' subcutaneous, skin and muscle from different depth. nuclide migration path emerged in these participants who did not appear any feeling like Deqi after subcutaneous injection [9]. Also another animal research found that the analgesic mechanism of WAA was related to neurohumoral regulation [10].

It seems that WAA is the first and perfect choice for the treatment of pain. And there are many acupuncturists using WAA in clinic and they also think that WAA is effective for the treatment of pain, but it is found that the evidence to support the effect and safety of WAA for pain is rare and few related researches reported in foreign literature. Therefore this systematic review and meta-analysis were conducted to confirm the safety issue and efficacy of WAA for pain in order to explore whether it has the value of clinical promotion.

## 2. Materials and Methods

### 2.1. Study Selection

This study searched the following databases without any language restriction: PubMed, Cochrane Central Register of Controlled Trials, ISI Web of Science, Scopus, CINAHL Plus (EBSCO), and Complementary Medicine. Searching of Chinese databases was also conducted which include China Journal Full-Text Database, China National Knowledge Infrastructure (CNKI), and Chinese Scientific Journal Database. Publications available from the inception of databases to December 12, 2012, were reviewed to find the appropriate randomized control trials of WAA for pain. WAA related terms (Wrist-Ankle Acupuncture, Wrist Acupuncture, Ankle Acupuncture, and Acupuncture) and pain related terms (pain, ache, soreness, analgesia, acesodyne, and pain-relieving) were used as keywords in English digital databases. The following keywords were used in Chinese digital databases: “Wanhuaizhen” (which means “WAA”) and “Tong” (which means “pain”). Two reviewers manually searched the reference lists of all retried trials and previous reviews and also hand-searched relevant conference proceedings and abstracts, on-going and unpublished studies, grey literature, and peer-reviewed journals.

### 2.2. Inclusion Criteria

All studies that meet the following conditions were included: (1) studies were randomized controlled clinical trials (RCTs); (2) WAA was used in the treatment group, WM, SA or BA was used in the control group; (3) participants suffered from pain symptoms; (4) studies had one or two following measurements of pain relief:one was pain score such as visual analogue scale (VAS), verbal rating scale (VRS), or numeric rating scale (NRS), and another was effective rate (ER).There were no restrictions in the category of pain, the cause of pain, and disease duration.

### 2.3. Exclusion Criteria

Studies which have one or more following conditions were excluded: (1) studies that are non-randomized trials; (2) studies did not use WAA as the major treatment; (3) studies that had repeated reporting with same results; (4) studies had incomplete data; (5) studies didn't set up pain measurement like pain scale or effective rate.

### 2.4. Study Characteristics

Two authors searched the databases and screened all citations independently (L. Zhu and P. Tin): (1) authors names and details of participants (e.g., age, gender, pain score, and which disease caused pain); (2) blinding, trial design, total sample size and each group sample size, intervention procedures, and followup; (3) pain score before treatment and after treatment if available, effective rate, and adverse events. If the two reviewers differed in their decision to include a study, disagreement was resolved by discussion. If the consensus still cannot be achieved after discussing, we would seek a third party for advising (L. Li). Excluding reasons were also listed in this study. When disagreements were associated with either the design of publications or the outcomes of trials such as not reporting safety issue and didn't conduct follow up, corresponding authors were contacted to confirm the data that we extracted from their publications or to clarify any ambiguity via email or telephone.

### 2.5. Risk of Bias within Studies

Two reviewers assessed the quality of the methods in included studies by using the Jadad score [18]. The Jadad scale for assessing the quality of RCT major included three criteria: “Randomization,” “Blinding,” and “Withdrawals and Drop-Outs.” The Jadad score ranged from 0 to 5 points; RCTs were classified as eligible in this study when they got a score of three or more. Two reviewers resolved the disagreements which were associated with methodological quality of these studies by discussion.

### 2.6. Statistical Analysis

All statistical analyses were performed using the Cochrane Collaboration Review Manage software (RevMan 5.1). Relative risk (RR) or risk difference (RD) with 95% confidence intervals (CI) was used for dichotomous outcomes (e.g., effective rate of pain relief and adverse events), while standardized mean differences (SMD), with 95% CI, were used for continuous outcomes (e.g., pain score). Only studies with a Jadad score ≥3 were included for meta-analysis of effective rate, pain score, and adverse events. *I* ^2^ statistics was used to assess the heterogeneity in included studies. Fixed-effect model was used if heterogeneity was insignificant (*I* ^2^ statistics ≤40%). Random-effect model was used when heterogeneity was statistically significant (*I* ^2^ statistics ≥40%). Sensitivity analysis would be conducted to assess the robustness of pooled outcomes and conclusions for this system review if the heterogeneity (*I* ^2^ > 50%) was still in a moderate or high level even when we used a random-effect model [13, 19].

## 3. Results

### 3.1. Study Selection

**Figure 1:**
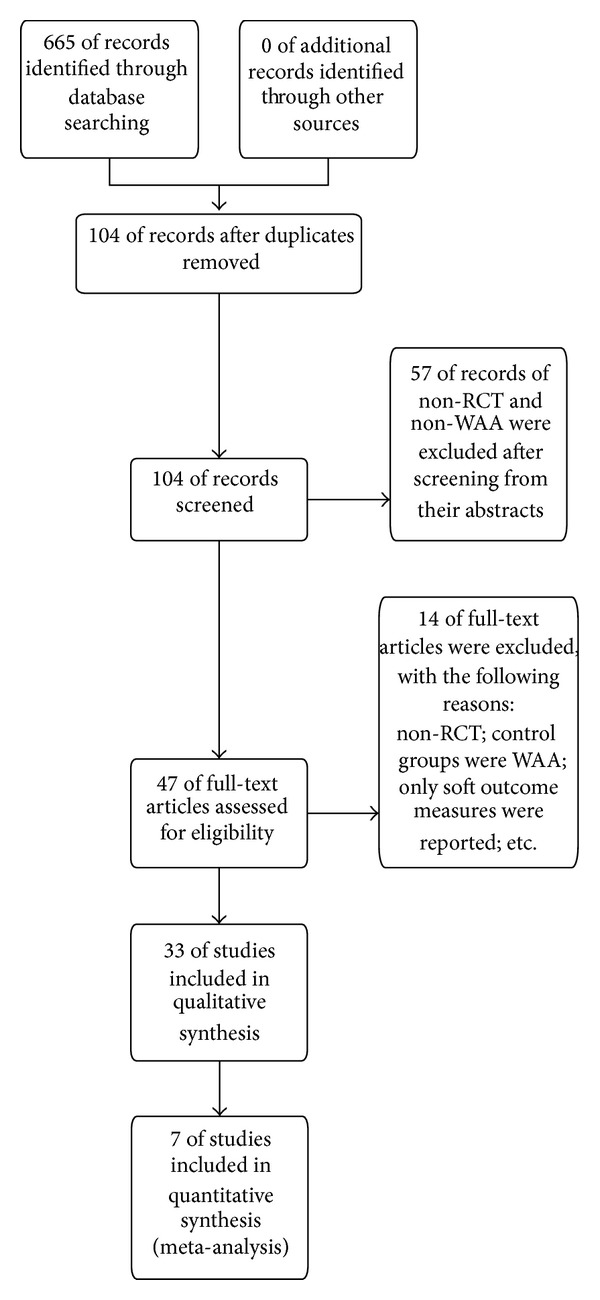
Flow diagram of the systematic review.

An initial search of RCTs yielded 665 potential literature citations (Figure 1). After screening, 437 articles were excluded because they were duplications or irrelevant records. The full texts of 228 articles were examined in detail to assess their relevance. Another 181 articles were excluded: 75 for search overlap and 98 for nonrandomized trials, 5 were not mainly about pain symptoms, and 3 did not mainly use WAA as treatment. As a result, 47 potentially appropriate RCTs were left. In the 47 potentially appropriate RCTs, 14 more studies were excluded, 2 of which contained duplicate experiments, 8 had neither pain score nor effective rate, and 4 were a comparison among the same group. Finally, 33 trials representing 3598 various patients suffering from pain met the inclusion criteria. Seven studies that represented moderate to high quality studies (Jadad score ≥3) were included for meta-analysis, in which adverse events and efficacy of WAA for pain were examined. Details of the excluded trials and the reasons for exclusion are available upon request from the authors.

### 3.2. Overall Study Characteristics

The 7 RCTs included a total of 723 participants with pain symptoms: 360 in WAA group and 363 in control group (e.g., body acupuncture, sham acupuncture, or western medicine). Among these 7 articles, 6 studies were published in Chinese and were conducted in Mainland China. The remaining 1 study was published in English and was conducted in Hong Kong. Western medicine was adopted as a control in 4 studies, 2 trials used sham acupuncture, and 1 study used body acupuncture as control group. The mean pain score at baseline was 5.94 in WAA group and 5.60 in control group. Pain score was measured at various time points (e.g., measured immediately after treatment or after 0.5 h, 1 h, 24 h, 48 h, and 1 month of the last treatment), with various methods, including NRS and VAS. Only 4 studies reported side events and 3 studies set up followup (Table 1).

### 3.3. WAA Treatment and Control Characteristics

Study 1 conducted a 3-parallel-arm trial for the treatment of root pain in lumbar disc prolapse. In this study WAA was the treatment group, and body acupuncture and western medicine were set up as control groups, respectively; only one time treatment was provided for all patients. Study 2 chose WAA (treatment group) and sham acupuncture (control group) for the treatment of acute lumbago, and also only time treatment was provided. Study 3 was conducted for chronic neck pain. In this study, electrical acustimulation of WAA was used in the treatment group and sham acupuncture was used in control group, 2 times treatment per week and totally 4 weeks treatment were provided for each group. Study 4 chose WAA combined with puncture bleeding and quick cupping (treatment group) and western medicine (control group) for herpes zoster; 1 time treatment per day, 3 days as a course and totally 3 courses treatment were provided for all participants. Study 5 was conducted for postherpetic neuralgia. In this study WAA was set up as treatment group and body acupuncture as control group, 1 time treatment per day, 5 days as a course, and totally 3 courses treatment were provided for each group, and patients relaxed 2 days between courses. Study 6 was conducted for pain after knee arthroplasty; WAA plus auricular plaster was the treatment group and western medicine was the control group. This study provided 1 time treatment per day, 10 days as a course, and totally 3 courses treatment. Study 7 was conducted for middle-late liver cancer pain, and WAA was used in treatment group and western medicine in control group. 1 time treatment per day and a total of 10 days of treatment were provided for each group [11, 12, 14–17, 20] (Table 1).

### 3.4. Efficacy Assessment

Study 1, study 3, study 4, study 5, study 6, and study 7 used effective rates as outcome assessment. But these six studies have different definition of effect rate; hence this meta-analysis combined their common characteristic in the definition of effective rate and finally defined effective rate as at least 30% pain reduction after treatment. In study 1, effective rate was 19.14% in WAA group, 1.32% in body acupuncture group, and 2.78% in western medicine group [11]. In study 3, 40% of participants in WAA group reported a reduction of numerical rating scale (NRS) ≥50% and 12.5% in sham acupuncture group; and 70% in WAA group reported a reduction of NRS ≥30% and 12.5% in sham acupuncture group [12]. In study 4, the effective rate was 97.5% in WAA group and 78.9% in western medicine group [20]. In study 5, the effective rate was 63.3% in WAA group and 40% in western medicine group [15]. In study 6, the effective rate was 95% in WAA group and 77.50% in western medicine group [16]. In study 7, the effective rate was 86.1% in WAA group and 92% in western medicine group [17]. VAS and NRS were used as the subjective measurements in four studies (1, 2, 3, and 6) [11, 12, 14, 16]. In study 1, the baseline VAS score was 7.00 ± 0.02 in WAA group, 6.80 ± 0.00 in body acupuncture group, and 7.10 ± 0.00 in western medicine group. Study 1 did not show the VAS score after treatment but only provided the percentage of pain reduction. Hence study 1 was not included in the meta-analysis of pain score. In study 2, VAS score in baseline was 5.383 ± 0.959 in WAA group and 5.343 ± 0.934 in sham acupuncture group; and VAS score after treatment was 3.530 ± 1.089 in WAA group and 4.673 ± 0.926 in sham acupuncture group. In study 3, NRS score in baseline was 6.50 ± 1.98 in WAA group and 5.87 ± 1.29 in sham acupuncture group; and NRS score after treatment was 3.61 ± 1.98 in WAA group and 5.35 ± 2.15 in sham acupuncture group. Study 6 did not provide the baseline VAS score but provided VAS score after treatment, which was 1.04 ± 0.54 in WAA group and 2.08 ± 0.76 in western medicine group. Studies 2, 4, 5, and 6 only assessed the short-term effect of WAA, whereas study 1performed 2-day followup, study 3 performed one-month followup, and study 7 performed ten-day followup to observe the long-term effect of WAA in pain relief (Table 1).

### 3.5. Effective Rate

**Figure 2:**
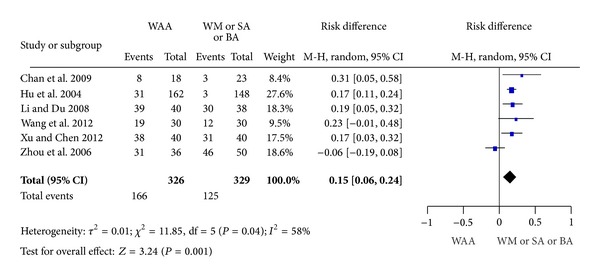
Effective rate.

This study conducted a meta-analysis of effective rate since six studies have the common efficacy assessment-effective rate. The WAA treatment arms achieved a significant efficacy as compared with western medicine, sham acupuncture, or body acupuncture in study 1, study 3, study 4, study 5, study 6, and study 7 (RD: 0.15, 95% CI: 0.06 to 0.24, *P* = 0.001), with heterogeneity (*P* = 0.04, *I* ^2^ = 58%) (Figure 2). It means that WAA was superior to western medicine, sham acupuncture, or body acupuncture in pain relief.

### 3.6. Pain Score

**Figure 3:**
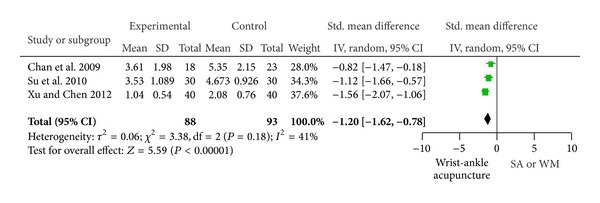
Pain score.

In the analysis of the change in pain score, the efficacy of WAA was found to be significant when compared with sham acupuncture and western medicine in study 2, study 3, and study 6 (SMD: −1.20, 95% CI: −1.62 to −0.78, and *P* < 0.00001), with heterogeneity (*P* = 0.18, *I* ^2^ = 41%) (Figure 3). This indicated that the efficacy of WAA was superior to western medicine and sham acupuncture in pain relief.

In this meta-analysis, it was found that the efficacy of WAA or WAA adjuvants was much better than western medicine, sham acupuncture, or body acupuncture.

### 3.7. Adverse Events Reporting

**Figure 4:**
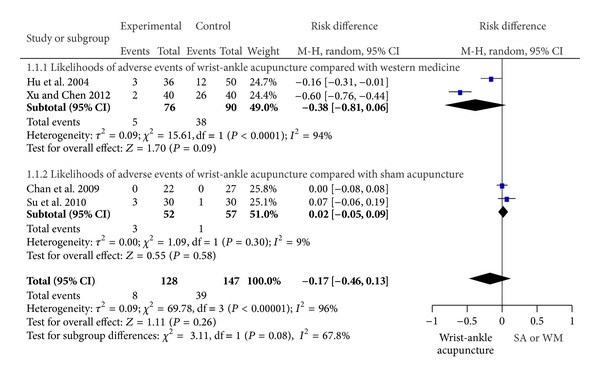
Likelihoods of adverse events of WAA compared with SA or WM.

Only four trials reported adverse effects (2, 3, 6, and 7). In studies 2 and 3, adverse events observed were temporary and not serious. These adverse events were usually subcutaneous hemorrhage from needle induced puncture wounds on the skin. The frequency of adverse events caused by WAA was nothing different from sham acupuncture [12, 14]. In study 6, 2 cases of adverse events were reported in treatment group (1 case of dizziness and 1 case of nausea), and 26 cases of side effects occurred in control group (7 cases of nausea, 6 cases of dizziness, and 13 cases of stomach problem) [16]. In study 7, adverse effects only occurred in patients suffering from severe pain and included one case of nausea and vomiting and two cases of vertigo [17] (Table 1). Overall, WAA was marginally safer than WM in the treatment of patients who suffered from pain (RD: −0.38, 95% CI: −0.81 to 0.06, and *P* = 0.09), while the heterogeneity was significant (*P* < 0.0001, *I* ^2^ = 94%). For safety issue, WAA was similar to sham acupuncture for the treatment of pain (*P* = 0.30, *I* ^2^ = 9%) (Figure 4).

## 4. Discussion

This is the first systematic review of wrist-ankle acupuncture versus sham acupuncture, body acupuncture, or western medicine for pain symptoms. All reviewers in this study have an educational background of Traditional Chinese Medicine and received high quality research training; especially Dr Li has a rich experience of training Master and Ph.D. and acupuncture clinic. Hence, all reviewers were competent to provide a high quality review and screening. This systematic review and meta-analysis were conducted according to PRISMA statement. In this meta-analysis, it was found that WAA or WAA adjuvant was much more effective than sham acupuncture, body acupuncture, and western medicine. Meanwhile WAA was associated with fewer side effects as compared with western medicine and equal safety to sham acupuncture. There is no doubt that it will be good news for patients suffering from pain symptoms and health care providers. Also, it will be a potential benefit for health policy makers because the application of WAA for the treatment of pain in clinic can largely decrease the side effects caused by using pain-killers and decrease medical cost due to the treatment of pain.

The majority of trials about WAA for the treatment of pain addressed the criteria concerning patients and experimental design, but the number of drop-outs and their reasons were rarely described in their studies. As a result, most studies scored less than 2 points on the Jadad scale (including 2 points) signifying a poorly designed study. This study attempted to contact the authors for more information regarding the drop-outs but very few responses were received. Although the random sequence generation in study 1 has a high risk of bias, the other details in the experimental design are rigorous. After discussion, we decided to include the study for meta-analysis. Combined with the data that the authors provided in response to our inquiries, seven RCTs were qualified which means these 7 studies received at least 3 scores.

Only studies 2 and 3 described the methods of blinding: both used single blinding. However, it is impossible that WAA practitioners be blinded to the treatments they provide in the clinical trials because the practitioners can distinguish nonacupoints and sham needles. Although studies 2 and 3 both used sham acupuncture for comparison, concerns still exist about whether sham needles can serve in randomized control trials and whether patients are really not aware of whether or not they underwent active acupuncture [21]. With the advancement of acupuncture in clinical trials, sham acupuncture has been used as a placebo in acupuncture clinical trials. Also, noninvasive sham acupuncture is easier to recognize in comparison with routine acupuncture than invasive sham acupuncture especially for those patients who have been previously treated with acupuncture [21–25]. However, the question of whether invasive acupuncture truly plays a nonactive role even in nonacupoints still remains because in traditional acupuncture treatment, many techniques exist, such as shallow insertion, that are similar to invasive sham acupuncture [26]. This is may be one of the reasons that even though noninvasive or invasive acupuncture has been around for many years in China, which is the origin of acupuncture, many acupuncturists still do not apply those techniques in the acupuncture clinical trials.

Since language restrictions in systematic reviews can influence results, we did not discriminate based on language [27]. However, the studies in our systematic review were almost all written in Chinese because many factors affect patients in choosing acupuncture as treatment method for pain in clinical: cultural background, geography, wealth, and available time. Meanwhile one study demonstrated that beliefs or expectations can exert a powerful influence on the effectiveness of acupuncture treatment [28]. There is no doubt that the Chinese most frequently receive acupuncture and also have the longest history of treatment with acupuncture. Hence, it is no surprise that they more willingly accept acupuncture. Moreover, WAA is a new subcutaneous needle therapy that is not as equally popular as traditional acupuncture in other locations. Naturally, WAA is less frequently accepted as a complementary therapy in other countries. This explains why almost all literature on WAA for pain-relief was written in Chinese.

Due to the small number of studies included and the heterogeneity across studies, we need to carefully treat the outcome of this meta-analysis. Adverse events were reported in only 4 studies, in which the frequency of adverse effects that occurred due to WAA was lower than western medication but equal to sham acupuncture. Moreover, these adverse events were generally subcutaneous hemorrhage from needle-punctured skin. Concerning the distinctive qualities and advantages of WAA, more cautious considerations should be given. Greater scrutiny is necessary in choosing control groups. Treatments such as noninvasive sham acupuncture may have an effect on patients and are not a sufficient negative control. Better control design is needed in the future.

## 5. Conclusion

Results from this meta-analysis provide evidence that WAA or WAA adjuvant helps patients relieve pain and is a quite safe therapy. Besides, WAA is a cheap and convenient treatment. Doubtless, it is good for the substantial number of people suffering from pain. Also, WAA deserves from health policy makers to pay more attention and it is worthy of clinical promotion. But this meta-analysis included relevant and rigorous RCTs are insufficient; hence, higher quality and more rigorously designed clinical trials with large enough sample sizes are needed to further confirm our findings.

## Figures and Tables

**(a):** 

Number	1st author (year)	Country	Disease category	Design (treatment group versus control group)	Sample size (treatment/control)	Adverse events
1 [11]	Zhou et al. 2006	China	Root pain in lumbar disc prolapse	3-parallel-arm (WAA versus BA; WM)	310 (162 + 76 + 72)	Not mentioned

2 [12]	Chan et al. 2010	China	Acute lumbago	2-parallel-arm (WAA versus SA)	60 (30 + 30)	WAA: 3 cases of subcutaneous hemorrhage SA: 1 case of punctured skin but not bleeding

3 [13]	Deeks et al. 2011	China	Chronic neck pain	2-parallel-arm (WAA + neck exercise versus SA + neck exercise)	49 (22 + 27)	Report: both AA and SA were safe and there were no adverse events

4 [14]	Su et al. 2010	China	Herpes zoster	2-parallel-arm (WAA + PB + QC versus WM)	78 (40 + 38)	Not mentioned

5 [15]	Wang 2012	China	Postherpetic neuralgia	2-parallel-arm (WAA versus BA)	60 (30 + 30)	Not mentioned

6 [16]	Xu 2012	China	Pain after knee arthroplasty	2-parallel-arm (WAA + AP versus WM)	80 (40 + 40)	Treatment group: 1 case of dizziness; 1 case of nausea Control group: 7 cases of nausea; 6 cases of dizziness; 13 cases of stomach problem

7 [17]	Hu 2004	China	Middle-late liver cancer pain	2-parallel-arm (WAA versus WM)	86 (36 + 50)	WAA: 1 case of nausea and vomiting; 2 cases of dizziness (total: 3) WM: 5 cases of nausea and vomiting; 5 cases of dizziness; 6 cases of constipation; 3 cases of drowsiness (total: 12)

**(b):** 

Followup	Measurement points	Outcome	Treatment course
2 days	After 0.5 h, 1 h, 24 h, and 48 h of treatment	Effective rate: 19.14% in WAA, 1.32% in BA, and 2.78% in WM	Only 1 time

No	Before 3 min of treatment and after 5, 10, 15, and 30 min of treatment	VAS: 35.30 ± 10.89 in WAA; 46.73 ± 9.26 in SA	Only 1 time

One-month	Immediately after treatment; 1 month after treatment	40% in WAA group reported a reduction of NRS ≥ 50% and 12.5% in SA group; 70% in WAA reported a reduction of NRS ≥ 30% and 12.5% in SA. NRS: 3.61 ± 1.98 in WAA; 5.35 ± 2.15 in SA	2 times/per week and a total of 4 weeks in both groups

No	After 1st, 2nd, and 3rd of treatment	Effective rate: 97.5% in WAA; 78.9% in WM	1 time/per day, 3 days as a course, and a total of 3 courses in both groups

No	After the last treatment	Total effective rate: 96.7% in WAA and 93.3% in WM; cure rate: 63.3% in WAA and 40% in WM	1 time/per day, 5 days as a course, a total of 3 courses; relaxation 2 days between courses

No	After treatment	VAS: 1.04 ± 0.54 in WAA and 2.08 ± 0.76 in WM; effective rate: 95% in WAA and 77.50% in WM	1 time/per day, 10 days as a course, and a total of 3 courses

10	After the last treatment	Effective rate: 86.1% in WAA and 92% in WM	1 time/per day; totally 10 days

Note: WAA: wrist-ankle acupuncture; WM: western medication; SA: sham acupuncture; NRS: numerical rating scale; VAS: visual analogue scale; BA: body acupuncture; QC: quick cupping; AP: auricular-plaster; PB: puncture bleeding.
